# The Flavonoid Agathisflavone Directs Brain Microglia/Macrophages to a Neuroprotective Anti-Inflammatory and Antioxidant State via Regulation of NLRP3 Inflammasome

**DOI:** 10.3390/pharmaceutics15051410

**Published:** 2023-05-05

**Authors:** Balbino Lino dos Santos, Cleonice Creusa dos Santos, Janaina R. P. Soares, Karina C. da Silva, Juciele Valeria R. de Oliveira, Gabriele S. Pereira, Fillipe M. de Araújo, Maria de Fátima D. Costa, Jorge Mauricio David, Victor Diogenes A. da Silva, Arthur Morgan Butt, Silvia Lima Costa

**Affiliations:** 1 Laboratory of Neurochemistry and Cellular Biology, Institute of Health Sciences, Federal University of Bahia, Av. Reitor Miguel Calmon S/N, Salvador 40231-300, Bahia, Brazil; balbino.lino@univasf.edu.br (B.L.d.S.);; 2 College of Nursing, Federal University of Vale do São Francisco, Petrolina 56304-917, Pernambuco, Brazil; 3 Group of Studies and Research for Health Development, University Salvador, Salvador 40140-110, Bahia, Brazil; 4 Department of General and Inorganic Chemistry, Institute of Chemistry, University Federal da Bahia, Salvador 40170-110, Bahia, Brazil; 5 School of Pharmacy and Biomedical Sciences, University of Portsmouth, Portsmouth PO1 2UP, UK

**Keywords:** anti-neuroinflammatory, agathisflavone, neuroprotection, NRLP3 inflammasome

## Abstract

Agathisflavone, purified from *Cenostigma pyramidale* (Tul.) has been shown to be neuroprotective in in vitro models of glutamate-induced excitotoxicity and inflammatory damage. However, the potential role of microglial regulation by agathisflavone in these neuroprotective effects is unclear. Here we investigated the effects of agathisflavone in microglia submitted to inflammatory stimulus in view of elucidating mechanisms of neuroprotection. Microglia isolated from cortices of newborn Wistar rats were exposed to *Escherichia coli* lipopolysaccharide (LPS, 1 µg/mL) and treated or not with agathisflavone (1 µM). Neuronal PC12 cells were exposed to a conditioned medium from microglia (MCM) treated or not with agathisflavone. We observed that LPS induced microglia to assume an activated inflammatory state (increased CD68, more rounded/amoeboid phenotype). However, most microglia exposed to LPS and agathisflavone, presented an anti-inflammatory profile (increased CD206 and branched-phenotype), associated with the reduction in NO, GSH mRNA for NRLP3 inflammasome, IL1-β, IL-6, IL-18, TNF, CCL5, and CCL2. Molecular docking also showed that agathisflavone bound at the NLRP3 NACTH inhibitory domain. Moreover, in PC12 cell cultures exposed to the MCM previously treated with the flavonoid most cells preserved neurites and increased expression of β-tubulin III. Thus, these data reinforce the anti-inflammatory activity and the neuroprotective effect of agathisflavone, effects associated with the control of NLRP3 inflammasome, standing out it as a promising molecule for the treatment or prevention of neurodegenerative diseases.

## 1. Introduction

Neuroinflammation orchestrated by glial cells, especially astrocytes, and microglia, is a fundamental element in the development of neurodegenerative diseases (NDD), such as multiple sclerosis and Alzheimer’s disease [1]. Microglia can assume distinct phenotypic states according to the microenvironmental stimulus to which they are exposed and may present an inflammatory or anti-inflammatory profile [2]. Hence, microglia are key for the progression of neurodegenerative diseases (NDD), since they are associated with the synthesis and secretion of cytokines and other mediators that contribute to both CNS damage and repair.

In vitro, studies have shown that the release of pro-inflammatory cytokines in microglial activation is dependent on the MAPKs and NF-κB signaling pathways, as well as on the activation of the NLRP3 inflammasome [1]. The NLRP3 inflammasome protein complex has been recognized for its crucial role in the innate immune response in neuroinflammatory processes, and its assembly requires the activation of the TLR4/NF-κB pathway, which promotes the expression of inflammatory cytokines such as IL-1β and IL-18 [2].

Flavonoids have shown in vitro neuroprotective properties, attenuating the activation of microglia exposed to harmful stimuli [3,4,5,6,7]. These molecules are a group of polyphenolic compounds, of low molecular weight and soluble in water. They comprise a group of metabolites originating from different plant species and exhibit a variety of biological activities, such as anti-inflammatory, antioxidant, antitumor, antimicrobial, and antiviral actions [4,6,7]. Studies show the potential of some flavonoids, such as icariin, fisetin, and rutin, to modulate inflammatory responses [3,4]. The biflavonoid agathisflavone present in *Poincianella pyramidalis* (Tul.) has been shown to be neuroprotective in in vitro models of glutamate-induced excitotoxicity and inflammatory damage [5,6,7]. However, the potential role of microglial regulation by agathisflavone in these neuroprotective effects is unclear. In this study, we analyzed the effect of conditioned medium from microglia treated with LPS and/or agathisflavone in PC12 cells to clarify the role of the anti-inflammatory effect of agathisflavone as a mechanism of neuroprotection.

## 2. Materials and Methods

### 2.1. Microglial Cell Cultures

Microglial cells were obtained from the cortex of newborn Wistar rats (0–2 days old). The animals were provided by the Animal Facilities of the Department of Physiology of the Institute of Health Sciences of the Federal University of Bahia (Salvador, BA, Brazil). All experiments were performed in accordance with the local Ethical Committee for Animal Experimentation of the Health Sciences Institute (CEUA protocol no 6731220818).

Isolation of microglia was performed according to the protocol established at the Guaza Laboratory at the Instituto Cajal in Madrid [8]. The brains of newborn Wistar rats were removed aseptically, meninges and blood vessels were removed from each cortex. Then the material was mechanically dissociated and filtered into a sterile 75 mm diameter Nitex membrane (R&D^®^). The filtrate was resuspended in DMEM medium (Island Biological Company- GIBICO^®^, in Grand Island, NY, USA), supplemented with 10% fetal bovine serum (FBS), 10% horse serum (HS), 4 mM L-glutamine, antibiotics (100 U/mL penicillin and 100 µg/mL streptomycin, Gibco^®^). The cells were cultured on poly-D-lysine (25 µg/mL) -coated flasks (TPP, Zellkultur, Switzerland) in a humidified atmosphere with 5% CO_2_ at 37 °C. Upon reaching confluence (7–10 days), adherent microglial cells were harvested by shaking at 165 rpm at 37 °C for 3 h. Isolated microglia were seeded into 96-, 24- or 6-well plates at a density of 3 × 10^4^/cm^2^; in 24-well plates, cells were grown under the surface of coverslips. The experiments were performed 24 h after plating. In all cases, the cells were cultured at 37 °C in 5% CO_2_.

### 2.2. Culture and Differentiation of PC12 Cells

PC12 are pheochromocytoma cells derived from the rat adrenal gland (*Rattus norvegicus*) and have been used for many years as an important in vitro model for neurons, because they exhibit the characteristic phenotype of neurons, such as the emission of long branched neurites and also cease mitotic activity, when stimulated with growth factors, such as neuronal growth factor (NGF) and fibroblast growth factor 1 (FGF1) [9,10]. In this study, PC12 cells were cultured in adherent Petri dishes containing DMEM medium (Gibco, Grand Island, NY, USA) supplemented with 10% FBS, 10% HS, 4 mM L-glutamine, and antibiotics (100 U/mL penicillin and 100 µg/mL streptomycin, Gibco^®^). The plates were kept in culture chambers at 37 °C and 5% CO_2_. When the cultures reached confluence, they were detached from the culture plates using a solution containing 0.05% trypsin and 0.02% EDTA in phosphate-buffered saline (PBS, 1X). After that, the cells were centrifuged at 1000 RPM, and seeded at a low concentration of 2 × 10^5^ cells per well in 24-well plates and incubated for 24 h. Then, for differentiation, the cells were treated with 100 ng/mL of NGF (Sigma-Aldrich, St. Louis, MO, USA) for 6 days. The NGF was dissolved in the culture medium, the medium being renewed every 48 h and maintaining the same concentration as the NGF. After this period of differentiation, the cells were treated with microglia conditioned medium (MCM).

### 2.3. Drugs and Treatments

Cell cultures were treated with the biflavonoid agathisflavone (FAB) extracted from *Cenostigma pyramidale* (Tul.) E. Gagnon & G. P. Lewis (syn: *Poincianella pyramidalis*, *Caesalpinia pyramidalis)*, as previously described [11]. The flavonoid was dissolved in dimethyl sulfoxide (DMSO, Sigma, St. Louis, MO, USA) at a stock concentration of 100 mM and stored at a temperature of 4 °C and protected from light. The concentration used in the treatment was 1 µM, after diluting in DMEM. To induce the inflammatory stimulus, treatment with *Escherichia coli* lipopolysaccharide (LPS) (Sigma Aldrich, Burlington, VT, USA) was performed, at a concentration of 1 µg/mL; final dilution was obtained at the time of treatment by diluting the stock solution directly into the fresh culture medium without FBS. For microglial cell treatment, the medium was replaced by a new culture medium containing 1 µM agathisflavone or LPS (1 µg/mL) or both flavonoid and LPS. The concentration and exposure time followed established protocols and took into account our previous studies concerning agathisflavone effects in vitro [5,6]. Control cultures were treated with DMSO diluted in a culture medium in a volume equivalent to agathisflavone concentration (0.01%). After 24 h of treatments, the microglial conditioned medium (MCM) from the control and treatment conditions (agathisflavone, LPS, and LPS with agathisflavone) were collected and used in the treatment of PC12 cells for 24 h. Experimental analyses were performed 24 h after treatments.

### 2.4. Cell Cytotoxicity Analysis

The MTT viability assay was used to assess cytotoxicity in microglia. For this, cells treated for 24 h with agathisflavone (1 µM) and/or LPS (1 µg/mL) or in the control condition (DMSO) were incubated with 3-(4,5-dimethylthiazole-2-yl bromide)-2,5-diphenyltetrazolium (MTT; Sigma). The cells were cultured in 96-well plates (Kasvi) for 24 h. After this period, the cultures were incubated with the MTT solution at a final concentration of 1 mg/mL for 2 h. Then, the solution of sodium dodecyl sulfate (SDS) at 20% (*w*/*v*) and dimethyl formamide (DMF) at 50% (*v*/*v*), at pH 4.7 to promote cell lysis. Then, the plates were kept at 37 °C overnight to dissolve formazan crystals. Cell viability was quantified by converting the yellow MTT into formazan to purple, promoted by mitochondrial dehydrogenases from living cells [12]. For this, the optical density of each sample was measured at 540 nm using a microplate reader. Three independent experiments were carried out for each analysis. The results were expressed as the percentage of viability of the treated groups in relation to the control, which was considered 100%.

### 2.5. Assessment of Reduced Glutathione Depletion

Reduced glutathione (GSH) is an important antioxidant compound for CNS cells, as it serves as a substrate for peroxidases and is conjugated with free radicals [13]. Here, we evaluated the protective effect of the flavonoid agathisflavone on LPS-induced cytotoxicity in primary microglia culture, through the determination of intracellular GSH. For this, the cells were seeded at a density of 3 × 10^4^/cm^2^, and after stimulation with LPS and treatment with agathisflavone, the cells were washed three times with PBS and incubated with 400 μL of medium containing 1 mM of monochlorobimane (MCB) (Sigma Aldrich, St. Louis, MO, USA) for 30 min in the dark. L-buthionine-S-R-sulfoximine (BSO) was used at a concentration of 1 mM as an inhibitor of GSH synthesis and adopted as a positive control. After the incubation time, the cells were washed again with PBS. The detection of free glutathione was performed using a fluorescence microscope (Leica, DFC7000, Wetzlar, Germany). Three independent experiments were carried out.

### 2.6. NO Production

Evaluation of the production of nitric oxide was carried out in samples of the cell culture medium, from the determination of the accumulation of sodium nitrite (NaNO_2_^−^) in the medium. For this, samples of medium were collected after treatment with LPS and/or agathisflavone at 1 µM. Equal volumes of culture medium and Griess reagent (1% sulfanilamide, 0.1% N-(1-naphthyl) ethylenediamine dihydrochloride, and 2% phosphoric acid; Sigma Aldrich, Saint Luis, MO, USA) were mixed. The mixture was incubated for 10 min at room temperature and then the absorbance was read on a 550 nm filter using a microplate reader (Varioskan—Flash Multimode Reader, ThermoPlate, Thermo Fisher Scientific, Inc., Vantaa, Finland). Three independent experiments were carried out.

### 2.7. Cell-Free Screening of Agathisflavone Antioxidant Activity DPPH• Free Radical Scavenging Test

The DPPH• radical scavenging assay was performed according to the method described by Blois (1958) [14] with some modifications for a 96-well microplate. The DPPH• radical stock solution was prepared in methanol to present an absorbance between 0.6 and 0.7 at 517 nm. Reaction volumes of 200 μL, containing 125 μM of DPPH• radical was prepared in methanol, and 50 μL of different concentrations of agathisflavone (1, 10, and 50 μM) were incubated at 25 ± 2 °C for 15 min and reading performed in the microplate reader (Varioskan—Flash Multimode Reader, ThermoPlate, Thermo Fisher Scientific, Inc., Vantaa, Finland) at a wavelength of 517 nm. This test was performed in the dark and at room temperature. The percentage of DPPH• inhibition was calculated by the following equation: % DPPH inhibition = 100 − [(sample Abs − blank Abs)/control Abs] × 100, where sample Abs is the flavonoid agathisflavone/trolox, blank Abs is methanol and Abs control DPPH• + 0.05% DMSO. Methanol was used as a negative control. Trolox (vitamin E analogue) was used as a positive control and treated under the same conditions as the sample. IC50 values denote the concentration of the sample, which is required to eliminate 50% of the DPPH radicals. Three independent experiments were carried out. 

### 2.8. Proliferation, Apoptosis, Morphology, and Cell Activation through Immunocytochemistry

The evaluation of microglial activation to LPS and/or treatment with flavonoid agathisflavone was investigated through immunocytochemistry using the following primary antibodies: anti-Iba1 (rabbit, 1:200; Wako, 019-19741, Saitama, Japan), anti-CD68 (rat, 1:100; Abcam, ab53444, Cambridge, United Kingdom), anti-CD206 (rat, 1:100, Bio-Rad, Hercules, CA, USA). Changes in the morphology of differentiated PC12 cells after treatment with microglia-conditioned medium, were evaluated by phase contrast microscopy and by immunocytochemistry for the cytoskeleton protein β-tubulin III (mouse, 1: 500; BioLegend, San Diego, CA, USA 801202) and the presence of apoptotic PC12 cells evaluated using anti-active-caspase-3 antibodies (rabbit, 1:300; Chemicon, ab3623 Darmstadt, Germany). Immunocytochemistry was performed in 24-well plates, with cells grown under the coverslip surface. After treatments, the culture medium was discarded, and the cells were washed three times with phosphate-buffered saline (PBS, Sigma) and then fixed with cold methanol for 20 min at room temperature (RT). Then, the cultures were washed three times with PBS and exposed to the primary antibodies diluted in PBS/BSA (1%) and kept in a humid chamber at 4 °C overnight. The next day, the cells were washed 3 times with PBS and then incubated with the following secondary antibodies diluted in PBS/BSA (1%): Alexa Fluor 488-conjugated goat anti-mouse IgG, (1:500, Life Technologies, Carlsbad, CA, USA), Alexa Fluor 594-conjugated goat anti-rabbit IgG (1:500, Life Technologies, Carlsbad, CA, USA). Incubation with secondary antibodies was kept under slow shaking for 2 h at RT and protected from light. After that, the cells were washed three times with PBS and incubated with 5.0 mg/mL of 4,6-diamidino-2-phenylindol (DAPI, Molecular Probes, Eugene, OR, USA), at RT for 10 min for nuclear staining. Then, the coverslips containing the cells were washed three times in PBS and mounted on slides containing the glycerol N-propyl-gallate solution (Sigma-Adrich, St. Louis, MO, USA). The experiments were carried out in triplicate. Quantification was analyzed using ImageJ 1.33u software (Wayne Rasband, National Institute of Health, United States). The images were observed and photographed using the fluorescence microscope (Leica, DFC7000).

### 2.9. Quantitative RT-PCR 

To evaluate gene expression for proteins of interest, after the treatment period, the culture medium was removed and then total RNA was extracted with Trizol^®^ reagent (Invitrogen, Waltham, MA, USA, Life Technologies, 15596026). Extraction was performed according to the manufacturer’s specifications. Total RNA purity and concentration were determined by spectrophotometric analysis using KASVI Nano Spectrum (cat# K23-0002). DNA contaminants were removed by treating the RNA samples with DNase using the Ambion DNA-free kit (cat# AM1906, Life Technologies™). For cDNA synthesis, SuperScript^®^ VILO™MasterMix (cat# MAN0004286, Invitrogen™, Life Technologies) was used in a 20-µL reaction with a concentration of 2.5 µg of total RNA, following the manufacturer’s instructions. Quantitative real-time PCR (qPCR) was performed using Taqman^®^ Gene Expression Assays (Applied Biosystems, CA, USA) containing two primers to amplify the sequence of interest, a specific Taqman^®^ MGB probe and TaqMan Universal Master Mix II with UNG (cat# 4440038 Invitrogen, Life Technologies™). The assays corresponding to the genes quantified in this study were IL1B (Rn00580432_m1), TNF Loc1036 (Rn01525859_m1), IL-6 (Rn01410330_m1), CCL2 (Rn00580555_m1), CCL5 (Rn00579590_m1), NRLP3 (Rn04244620). Real-time PCR was performed using the Quant Studio 7 Flex™Real Time PCR System (Applied Biosystems, CA, USA). The thermocycling conditions were performed according to the manufacturer’s specifications. The actin beta (ACTB) (Rn00667869_m1) and hypoxanthine phosphoribosyl transferase 1 (HPRT1) (Rn01527840_m1) targets were used as reference genes (endogenous controls) for normalization of gene expression data. Data were analyzed using the 2^−ΔΔCt^ method. The results represent the average of three independent experiments. 

### 2.10. Molecular Docking

To download the three-dimensional structure of the NLRP3 protein in pdb format, the database platform AlphaFold Protein Structure Database was used https://alphafold.ebi.ac.uk (accessed on 10 November 2022). Crystallographic structures from *Rattus norvegicus* and *Homo sapiens* without mutations were chosen. The agathisflavone ligand was downloaded on the platform ZINC15 www.zinc.docking.org (accessed on 10 November 2022) and MCC950 inhibitors were downloaded from PubChem https://pubchem.ncbi.nlm.nih.gov (accessed on 10 November 2022). The binders were downloaded in pdf format and then converted into pdb format through the program OpenBabel. AutoDockTools software was used to prepare protein and ligands. The water molecules were removed from the receptor and the polar hydrogens and Kollman charges were added... For the ligands, Gasteiger charges were added and then saved in pdbqt. After the preparation of the receptor and ligand, the gridbox was performed defining the binding site with the coordinates determined for the insertion of ligands. The NLRP3 binding site was determined in the NACTH domain, which is formed by amino acids 220 to 536, since studies have already proven the direct interaction of the MCC950 inhibitor with this domain.

The docking was performed by the AutoDockVina program using the command prompt programming language. In the end, the 9 best interactions of the ligand with each protein tested were provided, and the one that presented the lowest value of free energy of binding was chosen for analysis. The visualization and analysis of the interaction took place using the BIOVIA Discovery Studio Visualizer program. The flavonoid agathisflavone was defined as the ligand and the protein as the receptor. The analysis of the receptor-ligand interactions and then a 2D diagram was generated that made it possible to visualize the intermolecular bonds and all the amino acid residues present in the interaction between the receptor and the ligands, as well as to visualize the interaction in 3D structure.

### 2.11. Statistical Analyses

The results were analyzed by the GraphPad Prism 5.10 statistical program (San Diego, CA, USA) and recorded as median ± standard error of the means (SEM) of the evaluated parameters. To determine the statistical difference between the groups, an analysis of variance was performed using the OneWay ANOVA test, followed by the Student–Newmann–Keuls post-test for the parametric data. For nonparametric data, an analysis was performed using Kruskal-Walis and Dunns post-test. Confidence intervals were defined at a 95% confidence level (*p* < 0.05) was considered statistically significant). In all figures, error bars represent the SEM of at least three independent experiments.

## 3. Results

### 3.1. Agathisflavone Modulates the LPS-Induced Microglial Activation Profile 

**Figure 1:**
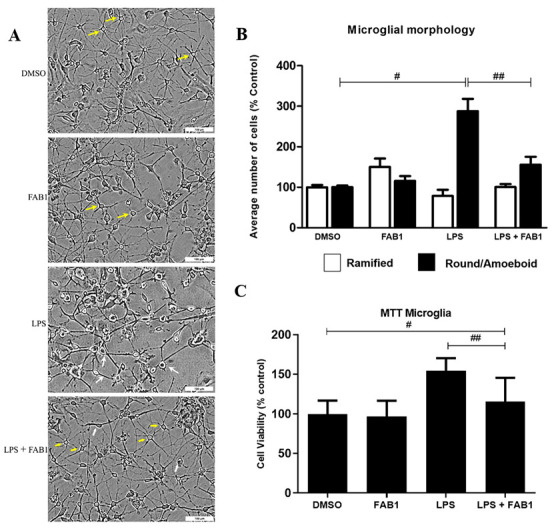
Effects of agathisflavone on LPS-treated microglia. Microglial cell cultures were treated for 24 h with vehicle control (0.001% DMSO), 1 µM agathisflavone (FAB1), LPS (1 µg/mL), or combined LPS plus FAB1. (**A**) Phase contrast photomicrographs of microglial cells in the different treatment groups, illustrating the ramified morphology (yellow arrows) and amoeboid morphology (white arrows); scale bar = 100 µm. (**B**) Quantification of microglia with ramified/branched or round/amoeboid morphology in the different treatment groups; results expressed relative to controls (100%) and tested for significance by one-way ANOVA; # or ## *p* < 0.05 (**C**) MTT analysis of microglial cell viability in the different treatment groups; results expressed relative to controls (100%) and tested for significance by one-way ANOVA; # or ## *p* < 0.05.

**Figure 2:**
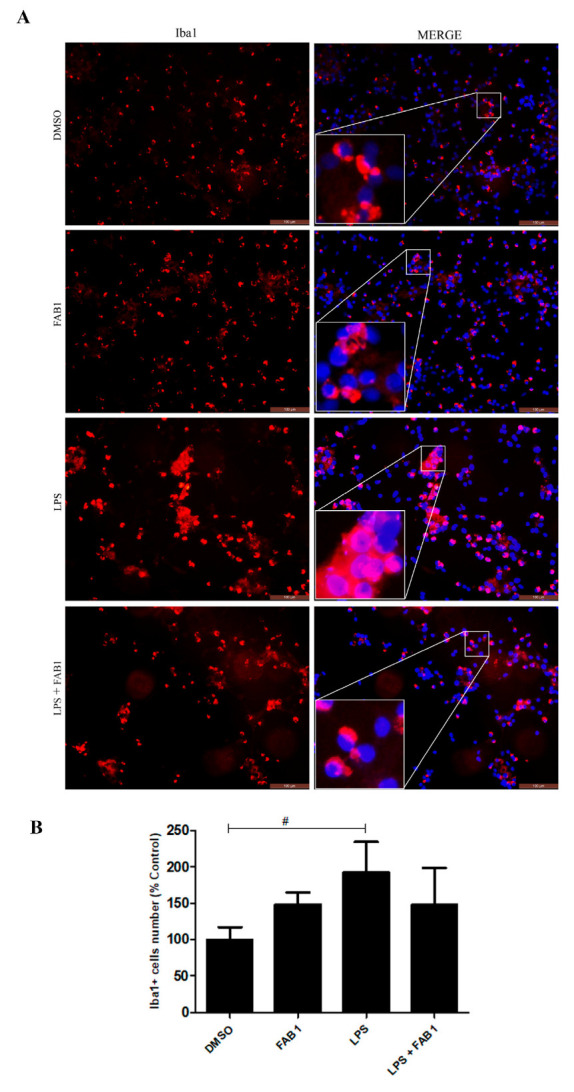
Effects of agathisflavone on Iba-1 expression in LPS-treated microglia. Microglial cell cultures were treated for 24 h with vehicle control (0.001% DMSO), 1 µM agathisflavone (FAB1), LPS (1 µg/mL), or combined LPS plus FAB1. (**A**) Immunostaining for Iba-1 (red) in microglial cultures in the different treatment groups; cell nuclei were stained with DAPI (blue). Scale bar = 100 µm. (**B**) The graph shows the quantification of Iba1+ cells expressed as a percentage of the total number of controls. Error bars represent mean ± SEM and were tested for significance by one-way ANOVA; # *p* < 0.05. The results are representative of repeated three independent experiments.

It is known LPS is capable of inducing microglia activation and inflammatory responses. Here, we investigated the effects of agathisflavone (FAB1) on microglia, alone and with LPS, to determine its effects on LPS-induced microglial activation; the DMSO vehicle (0.0001%) was used in control cultures. Firstly, to assess the effects of treatments on cell morphology and viability, we used the MTT test plus phase contrast microscopy (Figure 1), together with immunostaining for Iba-1 (Figure 2). In the control cultures and those treated with agathisflavone alone, microglia had a predominantly multipolar and branched morphology, with thin and long processes extending from small and rounded cell bodies (Figure 1A—some indicated by yellow arrows). In contrast, in LPS-treated cultures, microglia displayed a rounded or amoeboid morphology, with a reduction and retraction of their cytoplasmic processes, which were less numerous, shorter, and thicker (Figure 1A, some indicated by white arrows). These morphological changes were reduced by simultaneous treatment with LPS and agathisflavone and ramified microglia were more evident (Figure 1A, some indicated by yellow arrows). Quantification of microglial morphology confirmed these changes and demonstrated a significant increase in the relative density of amoeboid microglia following LPS treatment, which was significantly reduced by combined treatment with agathisflavone (Figure 1B). In addition, the MTT assay demonstrated a significant decrease in microglial cell viability following LPS treatment and this was significantly reduced by combined treatment with agathisflavone, which was not significantly different from control (Figure 1C).

The ionized calcium-binding adapter molecule 1 (Iba-1) is a calcium-binding protein specific for microglia/macrophages, being expressed both in vitro and in vivo. Compared with the control group (DMSO), immunofluorescence staining for Iba1 showed a significant increase following LPS treatment, but not following combined treatment with agathisflavone and LPS (Figure 2A,B). These changes are consistent with the cell counts presented above, which also showed an overall increase in microglial density following treatment with LPS, suggestive of microglial proliferation (Figure 2A,B).

**Figure 3:**
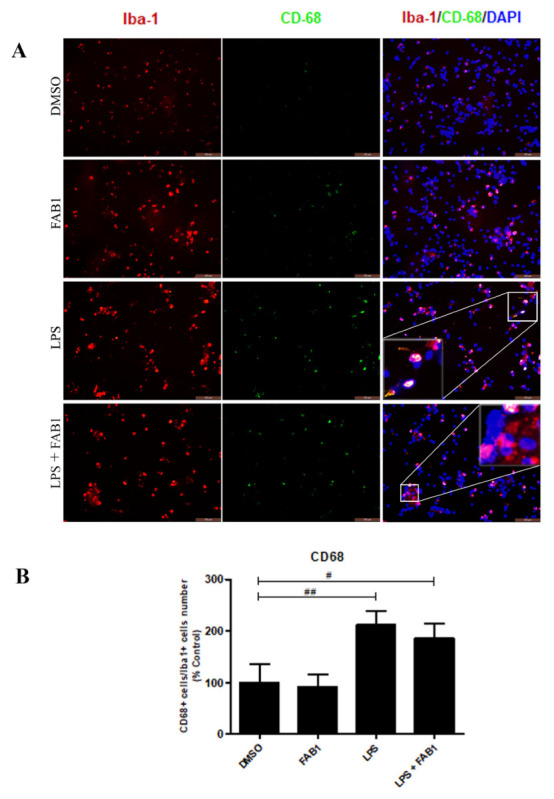
Effects of agathisflavone on CD68 expression in LPS-treated microglia. Microglial cell cultures were treated for 24 h with vehicle control (0.001% DMSO), 1 µM agathisflavone (FAB1), LPS (1 µg/mL), or combined LPS plus FAB1. (**A**) Immunocytochemistry of CD68 (green) with Iba1 (red) in microglial cultures in the different treatment groups; cell nuclei were stained with DAPI (blue). The results are representative of repeated three independent experiments. Scale bar = 100 µm. (**B**) Bar graphs show the percentage of CD68+/Iba-1+ cells in each condition; values are expressed as the mean ± SEM and were tested for significance by one-way ANOVA; # or ## *p* < 0.05.

**Figure 4:**
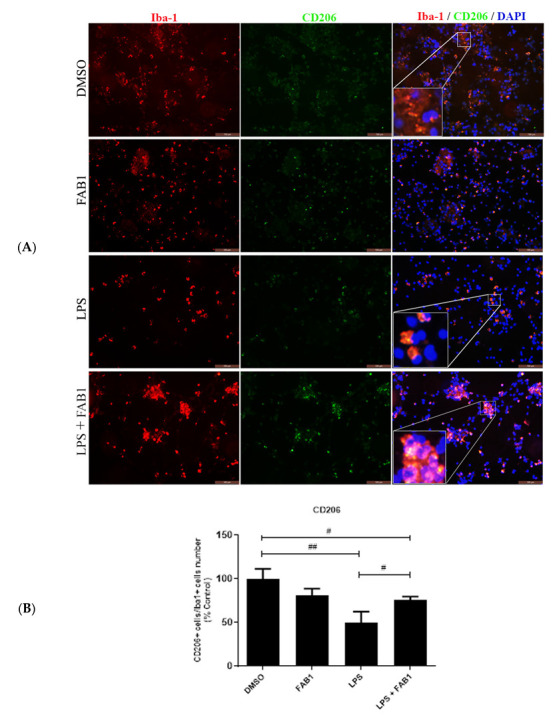
Effects of agathisflavone on CD206 expression in LPS-treated microglia. Microglial cell cultures were treated for 24 h with vehicle control (0.001% DMSO), 1 µM agathisflavone (FAB1), LPS (1 µg/mL), or combined LPS plus FAB1. (**A**) Immunocytochemistry of CD206 (green) with Iba1 (red) in microglial cultures in the different treatment groups; cell nuclei were stained with DAPI (blue). The results are representative of repeated three independent experiments. Scale bar = 100 µm. (**B**) Bar graphs show the percentage of CD206+/Iba-1+ cells in each condition; values are expressed as the mean ± SEM and were tested for significance by one-way ANOVA; # or ## *p* < 0.05.

Immunocytochemistry for the CD68 and CD206 proteins was performed to evaluate the effects of agathisflavone (1 µM) on the phenotypic profile assumed by LPS-treated microglial cells. CD68 is a transmembrane protein and CD206 (also known as mannose receptor C type 1 (MRC1) is a cell-surface protein that is used to distinguish between inflammatory states of activated microglia. Following treatment with LPS, we observed a significant increase in CD68 expression in microglia identified by Iba-1 immunostaining compared to control (Figure 3A,B). Combined treatment with LPS and agathisflavone also induced a significant increase in CD68 compared to control, but not compared to LPS treatment (Figure 3A,B). Expression of CD206 was significantly decreased in LPS compared to controls and this was partly reversed by combined treatment of LPS and agathisflavone (Figure 4A,B).

### 3.2. Agathisflavone Is an Effective Scavenger of Free Radicals and Has an Antioxidant Effect and Reduces NO Production in LPS-Treated Microglia

**Figure 5:**
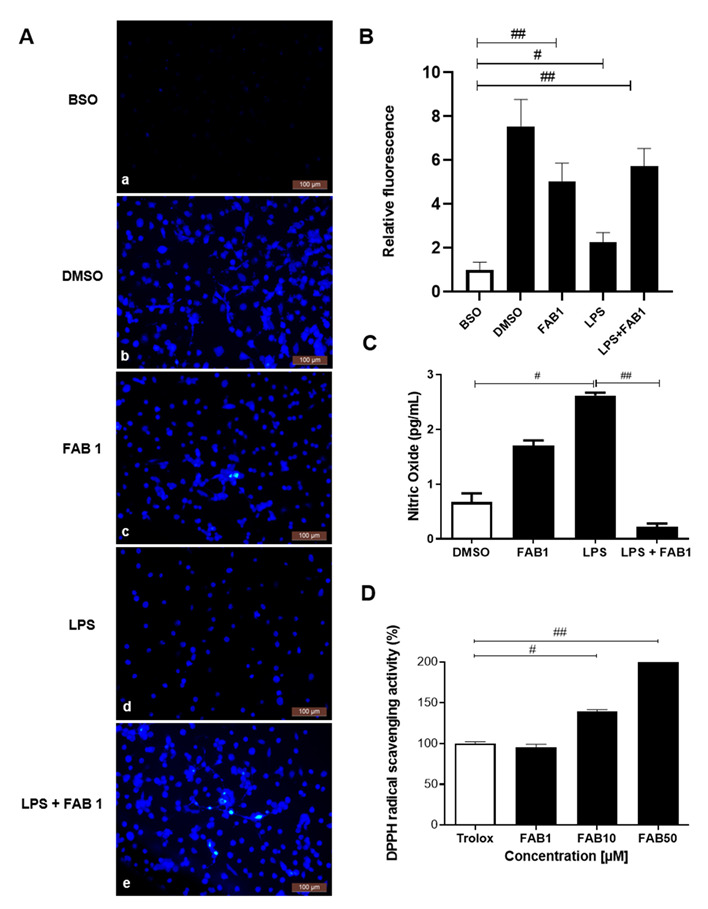
Effects of agathisflavone on GSH depletion and NO production in LPS-treated microglia. (**A**) Fluorescence measurement of glutathione (GSH) using the monochlorobimane (MCB) test in microglial cultures, treated with: (**a**) 1 μM D,L-buthionine-(S,R)-sulfoximine (BSO—positive control of the test); (**b**) 0.001% DMSO vehicle; (**c**) 1 µM agathisflavone (FAB1); (**d**) 1 µg/mL LPS; (**e**) combined treatment with LPS plus FAB1. Scale bars = 100 µm. (**B**) Graph showing fluorescence measurement of glutathione (GSH) using the monochlorobimane (MCB) test in microglial cultures, treated with: 1 μM D,L-buthionine-(S,R)-sulfoximine (BSO—positive control of the test); 0.001% DMSO vehicle; 1 µM agathisflavone (FAB1); 1 µg/mL LPS; combined treatment with LPS plus FAB1. Significance was assessed by the One Way ANOVA test followed by the Tukey test. Values are expressed as the mean SEM (n = 3). # *p* < 0.05 or ## *p* < 0.0001 (**C**) Graph showing the Griess reaction (NaNO2) for NO determination in microglial cultures incubated with 0.01% DMSO vehicle or treated with 1 µg/mL of LPS and/or 1 µM of agathisflavone (FAB1), for 24 h. Significance was assessed by the One Way ANOVA test followed by the Tukey test. Values are expressed as the mean SEM (n = 3). # or ## *p* < 0.05. (**D**) Activity of agathisflavone and trolox (standard) in scavenging the DPPH radical. The DPPH scavenging capacity of agathisflavone and the trolox control was compared over 15 min. Values are mean ± SEM (n = 2) # or ## *p* < 0.05 when compared to trolox (standard) for each concentration.

The monochlorobimane (MCB) test was used to assess intracellular GSH depletion by fluorescence microscopy. Treatment with BSO, a positive control for the technique, resulted in a marked reduction of GSH, and the same was observed in cells treated with LPS, whereas there was evident fluorescence in cultures treated with LPS plus agathisflavone, comparable to controls treated with vehicle DMSO (Figure 5A,B). Nitric oxide (NO) is produced by activated microglia in the presence of a stimulus to inflammatory damage, such as LPS [6], and so we evaluated this by measuring the accumulation of sodium nitrite (NaNO2-) in the medium after the different treatments. In cultures treated with LPS, there was a significant 3-fold increase in NO compared to the DMSO control group, and this was completely blocked by combined treatment with LPS and FAB1 (Figure 5C). The free radical scavenging activity of agathisflavone was determined using the assay for 2,2-diphenyl-1-picrylhydrazyl (DPPH), with trolox as a positive control. Agathisflavone had an IC50 of 10.27 μM, compared to 27.18 μM for Trolox (control), equivalent to a DPPH elimination capacity of 62.02% and 95.22% for trolox and agathisflavone, respectively (Figure 5D). 

### 3.3. Agathisflavone Regulates Gene Expression of Neuroinflammatory Mediators in LPS-Treated Microglia

**Figure 6:**
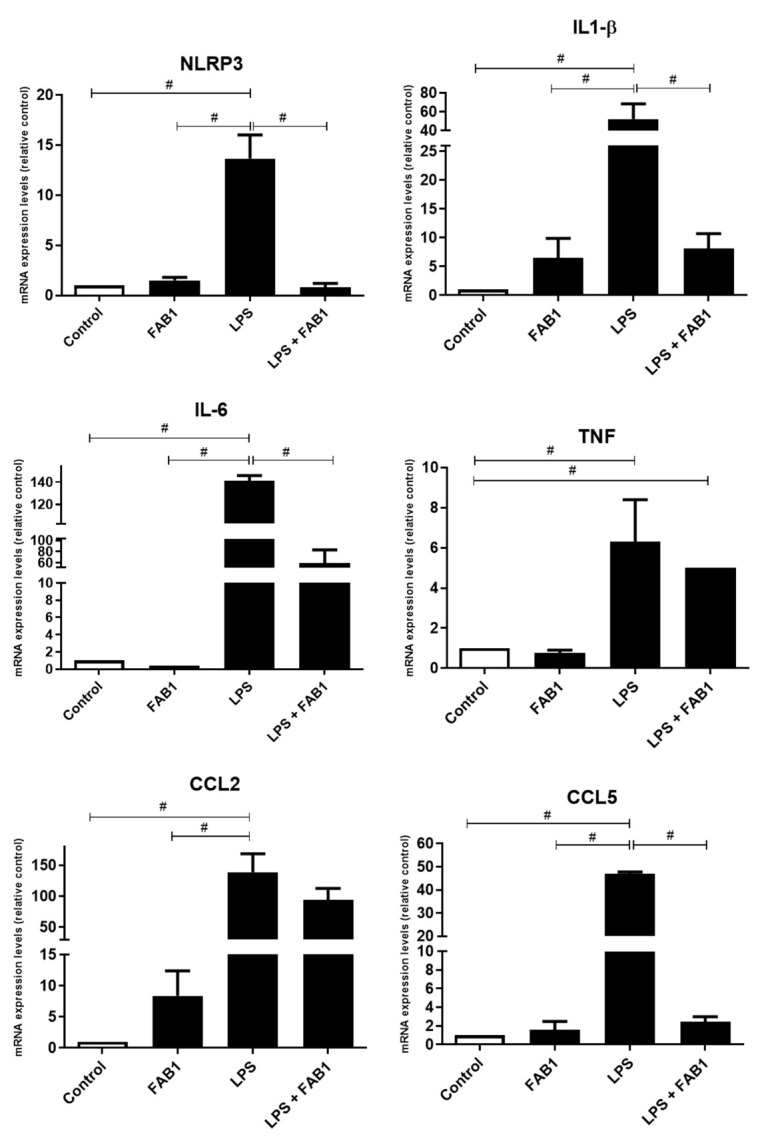
Effects of agathisflavone on inflammatory genes in LPS-treated microglia. Cultures of microglia were exposed to LPS (1 µg/mL) and/or treated with 1 µM agathisflavone (FAB1) for 24 h and RT-qPCR was performed on microglial cells to determine relative expression levels of mRNA for the component NLRP3 of the inflammasome complex, inflammatory cytokines IL1β, IL6, and TNF, and the chemokines CCL2 and CCL5. Data presented as mean ± SEM fold change relative to controls. Were tested for significance by one-way ANOVA; # *p* < 0.05 (n = 3).

In order to better characterize the anti-inflammatory effect of agathisflavone in microglial cultures exposed to LPS (1 µg/mL), RT-qPCR was used to measure mRNA expression of the following inflammatory mediators: interleukin 6 (IL-6), interleukin 1-β (IL1-β), tumor necrosis factor (TNF) and chemokines CCL5 and CCL2. In LPS-treated microglial cultures, there were significant increases in mRNA expression for IL-6, IL1-β, TNF, CCL2, and CCL5, when compared to controls (Figure 6). Combined treatment with agathisflavone significantly reduced mRNA expression for IL-6, IL1-β, and CCL5 compared to cultures treated with LPS alone; agathisflavone did not protect against the LPS-induced increases in TNF and CCL2 mRNA expression and did not completely protect against the increase in IL-6, which were significantly greater than controls. In addition, we evaluated the effect of agathisflavone in modulating the expression of the NOD-like receptor protein 3 (NLRP3) inflammasome, which corresponds to a protein complex associated with the activation of caspase-1 and the release of inflammatory cytokines such as Interleukin 1β (IL-1-β) and interleukin 18 (IL-18). Treatment of microglial cultures with LPS showed a significant increase in the expression of mRNA for NLRP3, which was completely blocked by combined treatment with LPS and agathisflavone, which was at control levels (Figure 6). The results indicate a general anti-inflammatory effect of agathisflavone on LPS-induced changes in microglia.

### 3.4. Agathisflavone Can Interact with NLRP3 Inflammasome 

**Figure 7:**
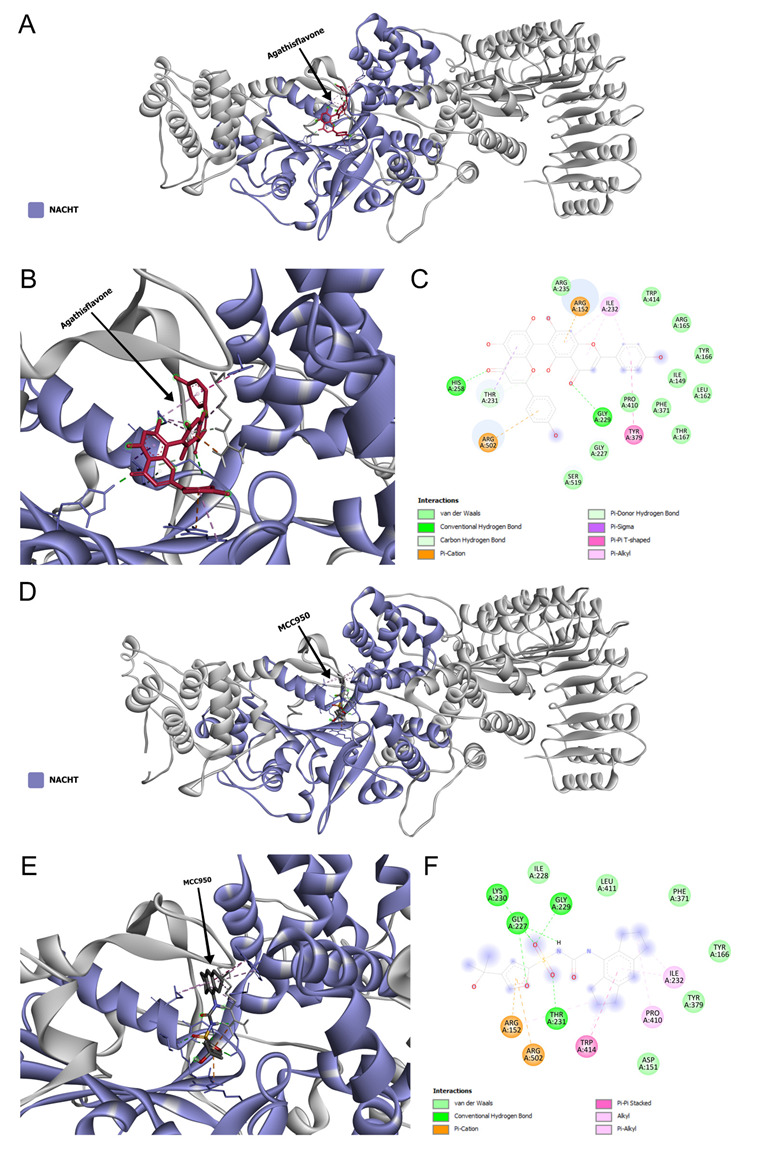
Agathisflavone and MCC950 interactions with the NACTH domain of NLRP3 inflammasome of *Rattus norvegicus*. (**A**) Visualization of complete NLRP3 structure with agathisflavone bounded. (**B**) Visualization of the NACHT region (in brown) with agathisflavone bounded. (**C**) 2D diagram showing amino acids and kinds of agathisflavone/NLRP3 interactions. (**D**) View of the complete NLRP3 structure linked to MCC950 (in red). (**E**) Visualization of the NACTH region (in brown) connected to MCC950. (**F**) 2D diagram demonstrating the amino acids and types of MCC950/NLRP3 interactions.

The results above demonstrated a highly potent dampening effect of agathisflavone on NLRP3 inflammasome and we, therefore, examined the potential interactions of agathisflavone in the NACHT domain of the NLRP3 inflammasome an important site for inflammasome ATPase activity required for its activation [15]. Docking analyses demonstrated the site of in silico interaction between these molecules (Figure 7A,B). The Gibbs free energy is used as a principle to analyze the spontaneity of bonds that occur in chemical reactions, the more negative the Gibbs free energy, the better the attraction between molecules. The binding between agathisflavone and the NACHT region of the NLRP3 of *Rattus novergicus* showed a Gibbs free energy value equivalent to −11.2 kcal/mol (Figure 7A). The NACHT domain of the NLRP3 of this species is composed of amino acids from position 218 to 534 and in it, we have the presence of two important motifs responsible for the activation of NLRP3, the motif walker A, and walker B. The interactions of agathisflavone in the NACTH domain of the species *Rattus novergicus* occurred through 22 interactions with the participation of 18 amino acids (Figure 7B,C), 11 van der Waal bonds, 2 conventional hydrogen bonds, 1 carbon–hydrogen bond, 2 Pi-cation bond, 3 Pi–Alkyl bond, 1 pi–donor hydrogen bond, 1 Pi–Sigma bond, and 1 Pi–Pi bond T-shaped (Figure 7C). Notably, we show that MCC950, a known inhibitor of NLRP3, also bound to the NACHT domain (Figure 7D,E), presenting a Gibbs-free energy equivalent to −9.8 kcal/mol with the participation of 15 amino acids, with 6 Van der Waal bonds, 5 conventional hydrogen bonds, 2 Pi-Cation bonds, 3 Alkyl bonds, 2 Pi–Alkyl bonds and 1 T-shaped Pi–Pi bond with MCC950 (Figure 7F). In addition, the interactions of equal amino acids between these two molecules are shown in Table 1.

**Figure 8:**
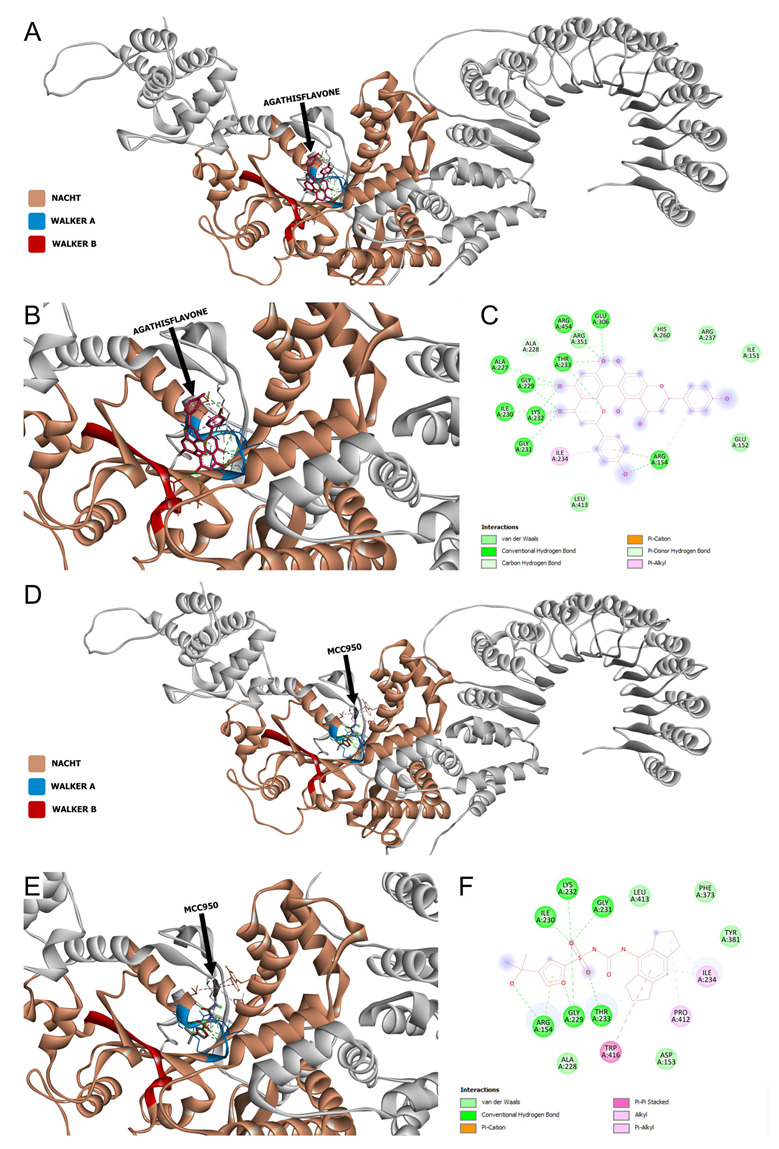
Agathisflavone and MCC950 interactions with the NACTH domain of NLRP3 inflammasome of *Homo sapiens*. (**A**) Visualization of complete NLRP3 structure with agathisflavone bounded. (**B**) Visualization of the NACHT region (in brown) with agathisflavone bounded. (**C**) 2D diagram showing amino acids and kinds of agathisflavone/NLRP3 interactions. (**D**) View of the complete NLRP3 structure linked to MCC950 (in red). (**E**) Visualization of the NACTH region (in brown) connected to MCC950. (**F**) 2D diagram demonstrating the amino acids and types of MCC950/NLRP3 interactions.

Docking performed with agathisflavone in the NACHT domain of the NLRP3 inflammasome of *H. sapiens*, also demonstrated the site of in silico interaction between these molecules (Figure 8A,B). Notably, we show that the binding between agathisflavone and the NACHT region of NLRP3 presented a Gibbs free energy value equivalent to −10.6 kcal/mol. The interaction of agathisflavone in the NACTH domain occurred both in the Walker A motif, with 15 interactions, and in the Walker B motif, with 2 interactions. The result of these agathisflavone interactions with NACHT-NLRP3 was the total participation of 17 amino acids, with 6 van der Waal bonds, 12 conventional hydrogen bonds, 2 carbon and hydrogen bonds, 1 Pi-cation bond, 3 Pi-Alkyl bonds and 2 pi-donor hydrogen bonds (Figure 8C). The MCC950, a known inhibitor of NLRP3, and its inhibition is associated with interactions with the walker A motif of the NACHT domain. Our in silico analysis showed that MCC950 also bound to the A walker motif of the NACHT domain (Figure 8D,E), presenting a Gibbs free energy equivalent to −9.7 kcal/mol with the participation of 16 amino acids, with 5 Van der Waal bonds, 7 conventional hydrogen bonds, 1 Pi-Cation bond, 5 Alkyl bonds and 2 Pi-Alkyl bonds with the MCC950 (Figure 8F). The interactions of equal amino acids between these two molecules are shown in Table 2. These results show that both agathisflavone and MCC950 bound at sites very close to this domain, suggesting possible inhibition of NLRP3 by the flavonoid.

### 3.5. Agathisflavone Modulates Microglia to a Neuroprotective Profile on LPS-Induced Toxicity

**Figure 9:**
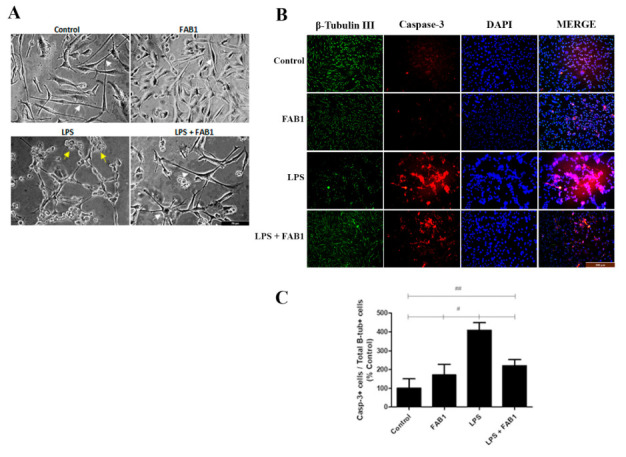
Agathisflavone has a neuroprotective effect on LPS-treated cells. PC12 cells were differentiated into neurons using NGF and treated with microglial conditioned medium (MCM) from microglial cultures treated with control vehicle (DMSO 0.01%), LPS (1 µg/mL), agathisflavone (FAB1, 1 µM) or combined LPS + FAB1. (**A**) Phase contrast microscopy of cells in the different treatment groups. In controls and FAB MCM cells had a typical neuronal morphology with multiple dendrites (white arrows), whereas cells appeared severely disrupted in MCM LPS (yellow arrows). Scale bars: 50 µm. (**B**) Immunocytochemistry with the neuronal marker B-tubulin III (green) and caspase-3 (red) to detect apoptotic cells, counterstained with the nuclear marker DAPI (blue). Scale bars: 100 µm. (**C**) Graph representing the proportion of cells double labeled with β-TubIII and caspase-3. Values are expressed as the mean ± SEM (n = 3) and were tested for significance by one-way ANOVA test. (# or ## *p* < 0.05).

The results above demonstrate that agathisflavone has an overall anti-inflammatory effect on LPS-treated microglia. As already reported, LPS is known for its ability to activate microglia and promote damage to neurons, through its recognition by toll-like receptors (TLRs) present in the microglial membrane, specifically TLR4, and consequent activation of the NFκB factor [16,17]. To determine whether the anti-inflammatory effects of agathisflavone on the microglial secretome are translated into neuroprotection, we determined the effects of microglial conditioned medium (MCM) on cultures of PC12 cells that had been differentiated into neurons with NGF, as confirmed by immunolabelling for β-tubulin III (Figure 8B). PC12 cells exposed to microglial conditioned medium (MCM) treated with vehicle (DMSO, controls), LPS (1 µg/mL), agathisflavone (1 µM), or both LPS and agathisflavone. The effects of MCM on PC12 cells were analyzed in terms of cell morphology by phase contrast microscopy (Figure 9A), and by immunocytochemistry for the neuronal marker β-tubulin III and apoptosis marker caspase-3 (Figure 9B). As shown in Figure 9A, PC12 cells exposed to MCM from controls (DMSO) or agathisflavone had a polarised morphology, with regular cell bodies and a network of long neurites. When exposed to MCM from LPS-treated microglia, PC12 cells displayed signs of cell damage, with swelling of the cell somata, formation of membranous globular structures, and loss of cellular neurites (Figure 9A, some indicated by yellow arrows). On the other hand, when PC12 cells were exposed to MCM from microglia treated with combined LPS and agathisflavone, the typical differentiated PC12 morphology was observed, with preservation of the cellular neurite network (Figure 9A, some indicated by white arrows). Equivalent results were observed using the neuronal marker β-tubulin III, where PC12 cells extended complex networks of neurites in MCM from controls, and this was severely disrupted in PC12 cultures treated with MCM from LPS-treated microglia, whereas neurites were preserved, and cells appeared as in controls in MCM from microglia treated with LPS plus agathisflavone (Figure 9B). In addition, there was intense immunolabelling for the caspase-3 marker in MCM treated with LPS, and this was significantly reduced in MCM from LPS and agathisflavone, when compared to the control (Figure 9B,C). The results demonstrate a cytoprotective effect of the secretome from agathisflavone treated microglial cultures.

## 4. Discussion

Microglia play an important role in defending the brain and repairing nervous tissue. In the injured or diseased brain, ramified microglia quickly change to an activated state, where they proliferate, migrate, change their morphology, express specific markers, release a variety of cytokines, and become phagocytic. In the present work, we used a model of LPS-induced neuroinflammation in primary cultures of microglia and demonstrated that biflavonoid agathisflavone modulates the microglial inflammatory response and promoted a neuroprotective inflammasome. In addition, we show that agathisflavone strongly interacts with the NACTH domain of NLRP3 inflammasome similar to the known inhibitor MCC950, suggesting this is an important mechanism of action of agathisflavone. 

As shown previously, LPS induced a robust microglial activation, as characterized by morphological changes, increased Iba-1 immunolabelling and changes in their cytokine/chemokine profile. A doubling of the number of Iba-1 positive microglia and overall increased cellular viability determined by the MTT assay indicated that LPS induced microglial proliferation, consistent with previous studies showing that Iba1 is positively regulated in microglial cells activated by CNS-damaging stimuli, participating in the modulation and reorganization of the cytoskeleton actin, contributing to cell membrane wrinkling, phagocytosis and in cell migration [18,19,20,21,22]. A key finding is that treatment with agathisflavone significantly inhibited these LPS-induced microglial reactive changes, as determined by cell morphology, number, and viability, which were not significantly different from control cultures. These results demonstrate that agathisflavone modulates microglial activation and proliferation. Upregulation in the expression of Iba1 also suggests the presence of active microglial cells. 

Another important effect observed in the present work was the changes in cell morphology and the expression of CD68 and CD206 proteins. As shown in our results, microglia in the presence of LPS showed a more amoeboid aspect, with shorter and thicker protractions, different from what was observed when the cells were exposed to the flavonoid, which showed a predominance of a multipolar and more branched phenotype. In addition, exposure to LPS induced an increase in CD68 expression and a reduction in CD206 expression and the percentage of CD206 positive cells, whereas, in LPS-stimulated cells treated with agathisflavone, there was an increase in CD206 expression, as well as an increase in the percentage of CD206 positive cells, although CD68 expression remained significantly higher than in controls, suggesting a complex effect of agathisflavone on microglia. This is consistent with changes in the expression patterns of cytokines/chemokines. Following LPS treatment the morphological changes in microglia to an activated amoeboid phenotype corresponded to increased expression of the pro-inflammatory cytokines/mediators IL-1β, IL-6, TNF-α, CCL2, CCL5, ROS, and NO [23,24]. These LPS-induced pro-inflammatory changes were completely inhibited by agathisflavone, with the exceptions of TNFα and CCL2 which were not reduced by agathisflavone, and IL-6, which was significantly reduced but remained higher than in controls. The results demonstrate that agathisflavone modulates LPS-induced microglial activation and promotes an overall anti-inflammatory phenotype.

Studies show that in the presence of harmful stimuli to brain tissue, such as LPS, microglia tend to assume a more amoeboid morphological profile, with retraction of its cell extensions and expression of cell surface molecules that recognize many of the inflammatory mediators present in the environment and that are associated with cell activation, migration, and proliferation. This morphological profile has been called by some authors as “amoeboid” microglia, resembling the so-called inflammatory profile of M1 macrophages, which is an oversimplification and does not adequately describe microglial activation states [5,7,25]. In inflammatory activation of microglia, studies show an increase in the expression of the CD68 marker [5,16,26]. CD68 is a transmembrane protein that has a molecular weight of 110 kDa, present in macrophages from various tissues and microglial cells in the CNS. The increase in CD68 expression appears to correlate with microglia activation, and its expression is regulated in response to various inflammatory stimuli, including the LPS toxin. The CD68 protein has a dotted distribution in resting microglial cell processes, whereas in activated cells of amoeboid morphology, its distribution becomes more evident [18,26]. 

Another activation state in which the microglia can also show amoeboid morphology is the M2a profile. In this case, the cell assumes anti-inflammatory characteristics, expressing on its surface markers such as arginase-1 (Arg-1), CD206, CD36, and CD163, as well as synthesizing cytokine IL-10 [16,24,27,28]. The increase in CD206 expression shown in our work corroborates the anti-inflammatory effect of flavonoids found by other authors [5,16,29]. The CD206 marker is a type C mannose and lectin receptor, expressed on the surface of macrophages/microglia where it acts as a pattern recognition receptor (PRR), helping these cells in the mechanisms of tissue repair, inflammation resolution, induction of immune tolerance and protection against excessive inflammation [30,31]. In this sense, we can infer the ability of agathisflavone to modulate the response to damage induced by LPS, for an anti-inflammatory profile. 

In the present study, we also evaluated the antioxidant effect of agathisflavone, through the glutathione depletion assay, NO dosage, and the DPPH elimination test. Reduced glutathione (GSH) has an antioxidant action in the brain, and its inhibition leads to the secretion of toxic products into central nervous system (CNS) cells, which may be related to neurological disorders [32,33]. Here, agathisflavone reduced GSH depletion in LPS-stimulated microglial cells. Lee et al. [32] showed in an in vitro model that the inhibition of γ-glutamylcysteine synthetase (BSO) induced a neuroinflammatory response in microglia and astrocyte cell lines, due to reduced intracellular levels of GSH. Work has suggested that oxidative stress leads to the active secretion of intracellular GSH in CNS cells, and that GSH depletion in neuroinflammation models leads to the activation of microglia, astrocytes, and consequent neuronal death [34,35].

Another important mediator in the neuroinflammatory process is nitric oxide (NO). In neuroinflammatory-related diseases, such as Alzheimer’s and Parkinson’s disease, activated glial cells increased the synthesis and the release of NO and other inflammatory mediators into the tissue microenvironment. In the present work, we observed that the flavonoid agathisflavone was able to negatively modulate NO synthesis. Studies have shown that NO is synthesized and released by activated microglia under the action of inflammatory stimuli, such as LPS, thus performing a neurotoxic action [5,36]. In activated microglia, in addition to the secretion of pro-inflammatory cytokines, there is also a generation of reactive oxygen species (ROS), nitric oxide (NO), and reactive nitrogen species (RNS). This set of mediators produced in microglia activation contributes to oxidative stress and neurotoxicity [37,38]. In a previous study carried out by our research group, the effect of agathisflavone in reducing nitric oxide production was shown in the primary coculture of neurons and glial cells [6]. Negative modulation of NO in microglia contributes to the reduction of neuroinflammation, consequently playing a neuroprotective role [37]. 

In the evaluation of the antioxidant properties, we showed that agathisflavone performed high activity in the scavenging of free radicals in a dose-dependent manner. Phenolic compounds are known for their antioxidant capacity by scavenging free radicals and reactive oxygen species [39,40]. Methanolic extracts from branches of *Juniperus macrocarpa*, which have a high amount of biflavonoids such as agathisflavone and amentoflavone, and show a high DPPH-reducing potential, showing inhibition of up to 80.23%, at a concentration of 1 mg/mL, when compared with ascorbic acid (87.29%), used as a reference [41]. Furthermore, studies have shown that molecules that have free hydroxyl groups, such as agathisflavone, are strongly linked with antioxidant activity through DPPH free radical stabilization by hydrogen radical donation [42]. 

In addition to the effects observed in microglial proliferation and morphology, agathisflavone also had an immunomodulatory effect, through the regulation promoted in the expression of mRNA of inflammatory mediators. In the present study, we show that agathisflavone reduced the expression of IL-6, IL1-β, TNF, CCL2, and CCL5 as well as the NLRP3 inflammasome complex. Many studies have also shown that polyphenols, especially flavonoids, are molecules that have interesting biological effects, including anti-inflammatory and neuroprotective effects [5,6,16,43,44,45,46,47,48,49,50,51]. In a study carried out by Monique et al. [6] it was shown that agathisflavone, similarly to our work, reduced the expression of inflammatory cytokines, such as IFNγ, TNFα, IL1β in LPS-stimulated microglia culture. In another study by Souza et al. [7], it was also shown that the flavonoid agathisflavone reduced the expression of the inflammatory cytokines IL-6, IL1β, and TNF, induced by glutamate in a model of neuron-glia co-cultures. Muhammad et al. [52], in a study carried out with BV-2 microglial cells stimulated with LPS, showed that the flavonoid hesperetin, a member of the subclass flavanone, which is found in citrus fruits such as oranges, significantly reduced levels of mRNA expression and production for the cytokines TNFα and IL1β, as well as reduced the production of IL-6. In another work carried out with BV-2 microglia stimulated with lipopolysaccharide (LPS), it was shown that pre-treatment with agathisflavone (5–20 μM) produced a significant reduction in the release of TNF-α, IL-6, IL-1β, NO and prostaglandin (PG) E2 [53].

An important feature of neurodegenerative processes is the increase in the production and release of inflammatory mediators, such as the cytokines IL-6, TNF, IL1β, and IL-18. In this process, there is a strong participation of astrocytes and microglia, in which, when activated as the result of the harmful stimulus the production of the described inflammatory mediators is induced, triggering, among others, a chronic inflammatory response and contributing to the progression of neurodegeneration. Many of these cytokines have their expression regulated by the signaling pathway such as that of the mitogen-activated protein kinase (MAPK), and studies have suggested that flavonoids may act by inhibiting this signaling pathway, by suppressing ERK1/2 phosphorylation and blocking activation by NF-kB [50,51,52]. Considering the expression of IL1β, studies have suggested that flavonoids interfere by blocking the activation of caspase-1 or in the organization of the NLRP3 inflammasome. The NLRP3 inflammasome is a multiprotein complex composed of an NLRP3 sensor, an ASC/PYCARD adapter, and caspase-1, which is essential for the activation of the cytokines IL1β and IL-18. In this sense, interferences in the organization of this protein complex consequently affect the activation of the inflammatory mediators IL1β and IL-18 [51]. 

As shown in our work, agathisflavone reduced the expression of the mRNA of NLRP3 inflammasome, thus suggesting that the tested flavonoid also interferes with the assembly of the inflammasome complex. In silico analysis showed that agathisflavone can interact with the NACHT domain, an essential site for NLRP3 activation, this interaction occurs in a similar way to the inhibitor of this molecule, MCC950. The NLRP3 sensor protein contains two motifs in the NACHT domain that are important for its ATPase activity, the Walker A motif for ATP binding and the Walker B motif required for ATPase activity [15]. Functionally, the presence of ATP has been shown to activate the complex and the ATPase activity of the Walker B motif inactivates NLRP3 [54,55]. Both motifs cited are highly conserved and mutations in its structure can compromise all inflammasome functions, indicating a key role of this region in the activation of the complex [15]. Given the closed conformation of the NACHT domain, a conformational change is necessary for the exchange of ADP in ATP and consequent activation of NLRP3 with the subsequent assembly of the NLRP3 inflammasome complex. It was observed that MCC950 can prevent the opening of the NACHT domain, through crucial links with Walker motifs A and B, stabilizing its closed form, that is, the inactive form of this domain [56]. This study shows through in silico analysis that agathisflavone can bind to these motifs as well as MCC950 and may interfere with the activation of the NLRP3 sensor protein, a key molecule to initiate the assembly of the NLRP3 inflammasome complex, which may be related to the control of mRNA expression for NLRP3 after flavonoid treatment. 

In the present work, we also indirectly evaluated the neuroprotective effect of agathisflavone in a model of neuronal cells obtained from the differentiation of cells of the PC12 strain. These cells, after being differentiated, were exposed to microglia conditioned medium. We observed that agathisflavone was able to preserve or restore morphological integrity as well as promote an increase in cells positive for β-tubulin III, and a reduction of caspase-3 positive cells. Class III β-tubulin is a protein present in the structural organization of neuronal cell microtubules [6,57,58,59]. Caspase-3 is a protease present in cells as an inactive proenzyme, which, when activated, is essential to the death process by apoptosis, through the specific cleavage of many important cellular proteins [60,61]. However, a study suggests that caspase-3 activation can also mediate non-apoptotic neuronal functions, such as differentiation of neural stem cells and consequent neurogenesis, as demonstrated in an in vitro model of neurospheres clonally derived from the striatum of murine embryos [62]. In this sense, our work shows that the reduction of caspase-3 positive cells may be associated with the ability of the flavonoid evaluated to reduce neuronal cell death induced by LPS. 

As previously discussed, many studies demonstrate that polyphenolic compounds are known for their biological antioxidant, neuroprotective and cognitive properties. In studies carried out by our group, the neuroprotective effect of the flavonoid agathisflavone has been shown. Souza et al. [7], for example, showed in primary neuron-glia co-culture, that treatment with the flavonoid agathisflavone promoted a neuroprotective effect against glutamate-mediated excitotoxicity as well as favoring an increase in the number of neurons through activation of estrogen receptors. In another study, Almeida et al. [6], also showed in neuron-glia co-culture obtained from the cerebral hemisphere of Wistar rats, that agathisflavone protected neurons from the inflammatory damage induced by LPS and IL-1β, as well as increasing the overall number of positive neurons for β-tubulin III and reducing of positive cells caspase-3, thus indicating, in addition to its neuroprotective effect, a possible neurogenic effect. In addition, studies have also shown the neurogenic effect of polyphenolic compounds in the hippocampus region. Kim et al. [63], for example, demonstrated in neural progenitor cells and in histological sections of the hippocampus of adult mice, that polyphenol curcumin was able to stimulate hippocampal neurogenesis and increase neural plasticity and tissue repair.

The effects observed in this study may be associated with the profile of secretome present in the conditioned medium from the microglia when treated with the flavonoid. As already discussed here, microglial cells exposed to harmful stimuli are induced to synthesize and secrete inflammatory mediators, and on the other hand, when treated with polyphenolic molecules, such as flavonoids, they can be induced to synthesize and secrete anti-inflammatory immune mediators in the environment. These molecules or mediators can be free in the microenvironment or inside extracellular vesicles (EVs), known as exosomes, and play their role in intercellular communication [64,65]. According to the literature, intercellular communication, such as that which occurs between neurons and glial cells, can be mediated through intercellular contact, through the action of molecules synthesized and secreted in the medium, or through EVs. The cells of the nervous system secrete EVs, which potentially transport proteins and RNA molecules (micro-RNAs and messenger RNAs), from one cell to another, contributing to signaling and the ability to modulate gene expression and to modify the phenotype of the cell target [64,66]. In microglia, for example, studies have shown the role of exosomes in the pathogenesis of neurodegenerative diseases. These vesicles can transport and release pathogenic proteins such as the β-amyloid peptide and other inflammatory signals that contribute to the regulation and expression of pro-inflammatory genes such as IL-1β, IL-6, inducible nitric oxide synthase and cyclooxygenase-2 in the recipient microglia [67,68]. 

The results present in this work clarify the role of the anti-inflammatory response in microglia induced by agathisflavone with its neuroprotective action, since agathisflavone down-regulates cytokines, cell surface markers and reactive species associated with pro-inflammatory microglia profile and conditioned medium from agathisflavone treated microglia is protective for PC12 cells. Thus, the results presented here reinforce the neuroimmunomodulatory and neuroprotective role of this flavonoid suggesting its potential as an adjuvant agent in the prevention and treatment of neurodegenerative disorders.

## Figures and Tables

**Table 1:** Comparison of amino acids and types of interactions of NLRP3-agathisflavone and NLRP3-MCC950 binding (NLRP3 *Rattus norvegicus*).

Amino Acids	Bonds MCC950	Bonds Agathisflavone
ARG: 152	1 Pi cátion	1 Van der Waals; 1 Alkyl
TYR: 166	1 Van der Waals	1 Van der Waals
GLY: 227	1 Van der Waals	3 conventional hydrogen
GLY: 229	1 conventional hydrogen	1 conventional hydrogen
THR: 231	1 hydrogen and carbon; 1 hydrogen pi-donor; 1 Pi-Sigma	1 conventional hydrogen
ILE: 232	3 Pi-Alkyl	1 Alkyl; 1 Pi-Alkyl
PHE: 371	1 Van der Waals	1 Van der Waals
TYR: 379	1 T-shaped pi-pi	1 Van der Waals
PRO: 410	1 Van der Waals	1 Alkyl
TRP: 414	1 Van der Waals	1 T-shaped pi-pi; 1 Pi-Alkyl
ARG: 502	1 Pi cátion	1 Pi cátion

**Table 2:** Comparison of amino acids and types of interactions of NLRP3-agathisflavone and NLRP3-MCC950 binding (NLRP3 *Homo sapiens*).

Amino Acids	Bonds MCC950	Bonds Agathisflavone
ARG: 454	1 Van der Waal	1 conventional hydrogen
GLU: 306	1 Van der Waal	1 conventional hydrogen
ARG: 154	1 conventional hydrogen; 1 Pi-cation; 1 Alkyl	1 conventional hydrogen; 1 Pi-cation; 1 Hydrogen Pi-donor
LEU 413	1 Van der Waal	1 Van der Waal
ILE: 234	1 Alkyl; 1 Pi-Alkyl	1 Pi-Alkyl
GLY: 231	1 conventional hydrogen	2 conventional hydrogens; 1 Pi-Alkyl
LYS 232	1 conventional hydrogen	2 conventional hydrogens; 1 Pi-Alkyl
ILE: 230	1 conventional hydrogen	1 conventional hydrogen
GLY: 229	2 conventional hydrogens	2 conventional hydrogens
ALA: 228	1 Van der Waal	1 Hydrogen-carbon; 1 Hydrogen Pi-donor
THR: 233	2 conventional hydrogens	2 conventional hydrogens; 1 Hydrogen-carbon

## Data Availability

Not applicable.
